# Anxiety, depression, and somatic symptom disorders in health care workers at high altitude during the rapid spread of the SARS-CoV-2 Omicron variant: A prospective cohort study

**DOI:** 10.3389/fpsyt.2022.1018391

**Published:** 2023-01-04

**Authors:** Xiaokai Feng, Chenlu Yang, Huanjuan Yang, Kai Wang, Yuanyuan Xu, Xiaoxia Zhang, Qiang Zhang, Juan Ma

**Affiliations:** ^1^Department of Respiratory and Critical Care Medicine, Beijing Institute of Respiratory Medicine, Beijing Chaoyang Hospital, Capital Medical University, Beijing, China; ^2^Department of Medical Service, Qinghai Provincial People’s Hospital, Xining, Qinghai, China; ^3^Department of Epidemiology and Biostatistics, Institute of Basic Medical Sciences, Chinese Academy of Medical Sciences, School of Basic Medicine of Peking Union Medical College, Beijing, China; ^4^Department of Neurosurgery, Qinghai Provincial People’s Hospital, Xining, Qinghai, China; ^5^Department of Gastroenterology and Hepatology, Guangdong Provincial People’s Hospital, Guangdong Academy of Medical Sciences, Guangzhou, Guangdong, China; ^6^Diagnosis and Treatment Center of High Altitude Digestive Disease, Xining Second People’s Hospital, Xining, Qinghai, China

**Keywords:** anxiety, depression, somatic symptom disorders, health care workers, high altitude, SARS-CoV-2 Omicron variant

## Abstract

**Objective:**

The ongoing spread of the Severe Acute Respiratory Syndrome Coronavirus 2 (SARS-CoV-2) Omicron variant and hypoxia exposure to high altitude are the susceptible factors of people’s psychological abnormalities, especially the health care workers (HCWs) in the front line of the epidemic. There is no dynamic observation data on the prevalence of mental health disorders among HCWs at high altitude. The study is to assess the prevalence of mental health outcomes and its influencing factors among HCWs at high altitude exposed to the SARS-CoV-2 Omicron variant.

**Methods:**

This prospective cohort study collected sociodemographic data and mental health measurements from 647 HCWs in 3 hospitals in Xining, Qinghai province from 13 April to 4 May 2022. After the mental health intervention for the above-mentioned people in the Chengdong district, we collected mental health indicators on days 7 and 14, respectively. We used the generalized linear model and the generalized estimation equation and for further analysis.

**Results:**

The baseline cross-sectional survey of 647 HCWs in the Chengdong and Chengbei districts of Xining, Qinghai province shows that the prevalence of depression, anxiety, and somatic disorders were 45.75, 46.52, and 52.55%, respectively. The multivariable model showed that chronic diseases and nucleic acid collection were associated with increased scores of GAD-7, PHQ-9, and PHQ-15. And the GAD-7 score of HCWs with elderly people at home increased by 0.92 points. Subsequent repeated measurements of the mental health of HCWs in Chengdong district in Xining, Qinghai province, showed that anxiety, depression, and somatic disorders were significantly relieved, and physical exercise showed a significant protective effect, while loans and nucleic acid collection showed an adverse effect after 2 weeks of intervention. Additionally, engaged in nucleic acid collection was the risk factor of anxiety and depression.

**Conclusion:**

In this survey of HCWs on frontline at high altitude during the rapid spread of the SARS-CoV-2 Omicron variant, participants reported experiencing mental health disorders, especially in those with chronic disease, loans, and those who worked with longer hours and engaged in nucleic acid collection in Xining, Qinghai province, China. Exercise may help to improve anxiety and physical disorders.

## Introduction

Many reports showed that public health disasters outbreaks generally cause substantial psychological effects (1–5). World Health Organization (WHO) cautioned that the Omicron variant of Severe Acute Respiratory Syndrome Coronavirus 2 (SARS-CoV-2) held a very high risk of infection (6). The ongoing spread of the SARS-CoV-2 Omicron variant is a cause of psychological stress which contributed to considerable depression and anxiety among the general population. A study showed that 16.5% of the population had moderate to severe depressive symptoms, 28.8% of the population had moderate to severe anxiety symptoms, and 8.1% of the population had moderate to severe stress levels in China (7).

It is particularly noteworthy that front-line health care workers (HCWs) during fighting against coronavirus disease 2019 (COVID-19) have been facing great pressure due to the high risk of infection, overwork, depression, isolation, lack of contact with family, and exhaustion, which may lead to a certain degree of mental health problems, such as stress, anxiety, depressive symptoms, insomnia, denial, anger, and fear (8–10). However, most of the current studies are cross-sectional studies, lacking dynamic observation of the changes in mental health (9, 11, 12). On the other hand, some studies have pointed out that altitude may also be a risk factor for mental health disorders (13–15). Especially exposure to increased altitude of residence is associated with relative hypobaric hypoxia, which could alter brain activity in multiple ways, for example, abnormal ratio of granulocytes to lymphocytes (14), reductions in serotonin production (16), and alterations in brain bioenergetics (17, 18), all these factors could contribute to depressive symptoms. So it is very necessary to dynamically observe the prevalence of mental health disorders among HCWs in high altitude areas and explore the factors associated with mental health disorders.

The main purpose of this study was to systematically and comprehensively describe the anxiety, depression, and somatic symptom disorders of HCWs working at high altitude during the rapid spread of SARS-CoV-2 Omicron variant. The second objective was to track the effect of psychological services, dynamically observe the changes of mental health of HCWs in high altitude area and explore related factors.

## Materials and methods

### Study design and participants

From 13 April to 4 May 2022, we used the online questionnaire managed by the web-based survey platform to survey HCWs who worked on frontline during the SARS-CoV-2 Omicron variant outbreak in Xining, Qinghai province, China. The participants of this study were selected based on conventional sampling, and we sent the quick response code (QR code) linked to the online questionnaire to the WeChat groups of three hospitals in Chengdong district and Chengbei district for questionnaire survey. We set that the questions in the questionnaire were required and could not be skipped, and collected the Internet Protocol (IP) address and the time spent filling in the questionnaire for quality control. We conducted psychological assessment at three time points: the first week, the second week, and the third week after joining the frontline of anti-epidemic. The respondents were those HCWs who have engaging in nucleic acid collection including doctors and nurses, as well as those who were administrative personnel and logisticians ever under medical training.

There were 647 HCWs joining the frontline from three hospitals participated in the first survey after a busy week in Qinghai, China. Among them, we excluded participants who took too short to complete the questionnaire (<250 s) (*n* = 14). Of those included, 288 (44.51%) HCWs worked in Chengdong district, 359 (55.49%) worked in Chengbei district. Unfortunately, SARS-CoV-2 Omicron infection was more serious in Chengdong district than Chengbei district. Therefore, the workload and intensity of HCWs in Chengdong district was almost twice that in Chengbei district. The anti-epidemic work ended shortly after the first investigation in Chengbei district, while there was still follow-up work in Chengdong district. In order to reduce psychological barriers and maintain the mental health of HCWs, some regular measures including meditation, online counseling, and psychotherapy interventions had been formulated and applied to HCWs in Chengdong district. Subsequently, 139 HCWs from the Chengdong district were given interventions and completed weekly assessments at the two follow-up surveys, as shown in Supplementary Figure 1.

### Background of working environment

We provided mental health intervention for all HCWs in the Chengdong district, and followed up the completed population. The detailed interventions are as follows. During the first week, the primary investigators provided the online Mindfulness-Based Stress Reduction specifically designed for HCWs, which contains 24 of audios according to the work and sleep patterns of HCWs. In addition, HCWs were encouraged to exercise for 10–30 min a day. A designated researcher supervised once a day to ensure the HCWs completed the meditation and physical exercise, and then encouraged the HCWs to share their feelings and discuss the benefits and challenges of meditation, tips to improve meditation practice, learn rejuvenation technique, learn bedtime prayer meditation, heartfulness practice overview, and formal questions and answers.

During the second week, the primary investigators invited a professional psychotherapist to teach the decompression and relaxation meditation course five times, and guide HCWs to practice abdominal deep breathing for 5 min and listen to music-relaxation for 20 min. Meditation practice asks participants to gently focus their attention, with eyes closed on the source of light within the heart. Rather than trying to visualize this, participants were asked to simply tune in to their hearts and be open to any experience that they may have. The same designated supervisor still instructs HCWs to practice and discuss after class every day.

During the third week, HCWs were arranged for home isolation. The same supervisor continued to online accompany HCWs every day, and encouraged them to keep to doing Meditation and/or Exercise so as to consolidate the training effect. HCWs back home firstly reflected on theirselves for the events of the day, and then did relaxation meditation for 15 min before falling asleep every night. Guided by the soothing music background, they can imagine that the boring pressure and heaviness were leaving the body through the back in the form of smoke or vapor. Then a flow of purity, lightness, and freshness was following filling their whole body.

Each individual could complete the questionnaire only once. Electronic informed written consent was obtained from all respondents before the data collection. Besides, the research was approved by the two Institutional Review Boards (IRBs) including Qinghai Provincial People’s Hospital (Ethical number: 2022-065) and Xining Second People’s Hospital (Ethical number: 2022017). The two IRBs thought that the research met the requirement of ethic and had important clinical value so as to be approved 1 week before the study.

### Measurements

The questionnaire consisted of the following two parts: sociodemographic characteristics including gender, age, qualification, educational background, marital status, occupation, household income, title, employment position, working years, experience of fight against COVID-19, whether engaged in nucleic acid collection, working hours per day, and booster injection, and a series of psychiatric questionnaires including “Generalized Anxiety Disorder 7-Item Scale (GAD-7),” “Patient Health Questionnaire-9 (PHQ-9),” and “Patient Health Questionnaire-15 (PHQ-15).” In the three scale cutoffs are mild (5–9), moderate (10–14), and severe (≥15), more detailed information about all these scales was described in Supplementary material.

### Generalized Anxiety Disorder 7-Item Scale

Generalized Anxiety Disorder 7-Item Scale is a self-rating measure used to assess general anxiety disorder, which consists of 7 items and has good reliability and validity as established measures of anxiety (19). Each item is scored on a four-point scale (0 = none at all; 1 = part of the time; 2 = more than half of the time; 3 = almost every day) to investigate the frequency of 7 situations in the past 2 weeks (Supplementary Table 1). A total score of ≤4 points indicates the absence of anxiety, 5–9 points indicates mild anxiety, 10–14 points indicates moderate anxiety, and ≥15 points indicate severe anxiety.

### Patient Health Questionnaire-9

Patient Health Questionnaire-9 is a self-rating measure used to assess depression and depression severity (20), which is the most widely used instrument for screening depression in primary health care (20, 21). Each of the 9 items is divided into four levels (0 = not at all; 1 = part of the time; 2 = more than half of the time; 3 = almost every day) to investigate the frequency of 9 items in the past 2 weeks and give scores (Supplementary Table 2). The total score ranged from 0 to 27. The study reported that it was of more clinical significance to use 10 as the cut-off points for depression symptoms (22). In the scale cutoffs for depression are mild (5–9), moderate (10–14), and severe (≥15).

### Patient Health Questionnaire-15

The PHQ-15 mainly evaluates the degree of difficulty caused by various common physical symptoms (23), which includes the following somatization symptoms: stomach pain, back pain, pain in arms and legs, pain during intercourse, headaches, chest pain, dizziness, heart race, short breath, constipation, nausea, gas, trouble sleeping, and feeling tired (Supplementary Table 3). Symptoms are assessed for the last 4 weeks on a three-point Likert-type scale ranging from 0 (not bothered at all), over 1 (bothered a little), to 2 (bothered a lot). The derived total scores thus range from 0 to 30. In the scale cutoffs for somatic distress are mild (5–9), moderate (10–14), and severe (≥15).

### Statistical analyses

Continuous variables were presented as median (P25 and P75) and categorical variables were presented as number and percentage (%). We used Student’s *t*-test to explore the difference of normally distributed continuous variables between HCWs in Chengdong and Chengbei district, and for non-normally distributed continuous variables, we used Mann–Whitney *U* test. The χ^2^ or Fisher’s exact test were used for categorical variables. The percentile bar chart shows the prevalence of anxiety, depression, and somatic symptom disorders estimated by different mental health scales and the distribution of severity. For the cross-section, we used the generalized linear model to explore the relationship between different scores of mental health scales and sociodemographic characteristics. For the part of repeated measurements after psychological intervention, we described the mental health scores at baseline, day 7 and day 14, and the prevalence of anxiety, depression, and somatic disorders of different severity with line chart and percentage bar chart. Additionally, we used the generalized estimation equation to explore the changes of anxiety, depression, and somatic symptoms disorders over time and their related factors, and used the forest map to display them. All statistical analyses were performed using the Statistical Analysis System, version 9.4 (SAS Institute, Cary, NC, USA) and R (version 3.5.3^1^). All tests were two-tailed, and *P* < 0.05 was considered statistically significant.

## Results

### Sociodemographic characteristics and mental health status of HCWs at baseline

Compared with the HCWs in Chengbei district, the proportion of HCWs in Chengdong district who were male, highly educated, injected with booster, had previous experience of fight against COVID-19, and engaged in nucleic acid collection was higher, and the proportion of nurses and HCWs who exercised was lower. In addition, the daily working hours in Chengdong district were longer. There was no statistical difference in the distribution of age, household income, loan, living alone, elderly, children, chronic diseases, GAD-7 score, PHQ-9 score, and PHQ-15 score of health workers in the two regions (Table 1).

**TABLE 1:** Baseline characteristics of Chengdong and Chengbei district.

Variables	All (*n* = 647)	District		*P*-value
		Chengdong (*n* = 288)	Chengbei(*n* = 359)	
Age (years)	31 (28, 35)	30 (27, 35)	31(28,35)	0.281
Males, *n* (%)	116 (17.9)	68 (23.6)	48(13.4)	<0.001
Married, *n* (%)	430 (66.5)	189 (65.6)	241(67.1)	0.687
Education, *n* (%)				<0.001
Below bachelor degree	42 (6.5)	30 (10.4)	12(3.34)	
Bachelor degree	547 (84.5)	245 (85.1)	302(84.1)	
Master degree and above	58 (9.0)	13 (4.51)	45(12.5)	
Nurse, *n* (%)	453 (70.0)	190 (66.0)	263(73.3)	0.044
Working years, *n* (%)				0.174
<5 years	175 (27.0)	90 (31.3)	85(23.7)	
5–9 years	263 (40.7)	113 (39.2)	150(41.8)	
10–19 years	174 (26.9)	70 (24.3)	104(29.0)	
≥20 years	35 (5.4)	15 (5.21)	20(5.57)	
Title, *n* (%)				0.094
Primary and below	436 (67.4)	204 (70.8)	232(64.6)	
Middle and above	211 (32.6)	84 (29.2)	127(35.4)	
Booster injection, *n* (%)	468 (72.3)	225 (78.1)	243(67.7)	0.003
Household income, *n* (%)				0.728
<10,000 ¥/month	416 (64.3)	190 (66.0)	226(63.0)	
10,000–19,999 ¥/month	186 (28.7)	79 (27.4)	107(29.8)	
≥20,000 ¥/month	45 (7.0)	19 (6.60)	26(7.24)	
Loans, *n* (%)	464 (71.7)	201 (69.8)	263(73.3)	0.330
Live alone, *n* (%)	172 (26.6)	75 (26.0)	97(27.0)	0.780
With kids, *n* (%)	354 (54.7)	150 (52.1)	204(56.8)	0.229
With elderly, *n* (%)	443 (68.5)	191 (66.3)	252(70.2)	0.292
With chronic disease, *n* (%)	86 (13.3)	38 (13.2)	48(13.4)	0.948
Exercise, *n* (%)	151 (23.3)	48 (16.7)	103(28.7)	<0.001
Previous experience of fight against COVID-19, *n* (%)	474 (73.3)	240 (83.3)	234(65.2)	<0.001
Engaged in nucleic acid collection, *n* (%)	493 (76.2)	232 (80.6)	261(72.7)	0.020
Working hours per day, *n* (%)				<0.001
<6 h	56 (8.7)	32 (11.1)	24(6.69)	
6–7 h	343 (53.0)	140 (48.6)	203(56.5)	
8–9 h	220 (34.0)	94 (32.6)	126(35.1)	
>9 h	28 (4.3)	22 (7.64)	6(1.67)	
GAD-7 score	4 (1, 7)	4 (1, 6)	4(1,7)	0.429
PHQ-9 score	4 (1, 8)	4 (1, 8)	4(1,8)	0.617
PHQ-15 score	5 (2, 9)	5 (2, 9)	5(2,9)	0.600

Data are presented as median (IQR), or *n* (%). *P*-values comparing Chengdong and Chengbei district were from a two-sample *t*-test, Mann–Whitney *U* test, χ^2^ test, or Fisher’s exact test. *P* < 0.05 was considered statistically significant. GAD-7, Generalized Anxiety Disorder 7-Item Scale; PHQ-9, Patient Health Questionnaire-9; PHQ-15, Patient Health Questionnaire-15; COVID-19, coronavirus disease 2019; IQR, interquartile range.

**FIGURE 1:**
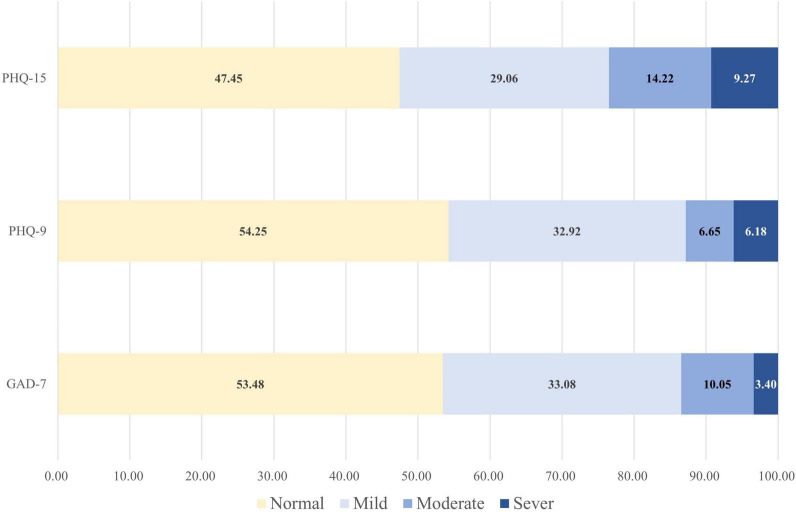
The prevalence of anxiety, depression, and somatic disorders of medical staff fighting against COVID-19 based on different scales. GAD-7, generalized Anxiety Disorder 7-Item Scale; PHQ-9, Patient Health Questionnaire-9; PHQ-15, Patient Health Questionnaire-15.

As indicated in Figure 1, the prevalence of anxiety (GAD-7 score ≥5), depression (PHQ-9 score ≥5), and somatic symptom disorders were (PHQ-15 score ≥5) 46.52% (95% CI: 42.68–50.37%), 45.75% (95% CI: 41.91–49.59%), and 52.55% (95% CI: 48.70–56.40%) in all participants, respectively.

### Association between psychological scores and sociodemographic characteristics at baseline

As shown in Table 2, compared with HCWs without elderly people at home, the GAD-7 score of HCWs with elderly people at home increased by 0.92 points (95% CI: 0.10–1.74, *P* = 0.028) on average. The average GAD-7 score of HCWs with chronic diseases increased by 1.46 points (95% CI: 0.45–2.46, *P* = 0.005). Moreover, HCWs who engaged in nucleic acid collection had a higher GAD-7 score (1.03, 95% CI: 0.19–1.87, *P* = 0.016). HCWs who had chronic disease (1.79, 95% CI: 0.65–2.93, *P* = 0.002) and who engaged in nucleic acid collection (1.20, 95% CI: 0.25–2.15, *P* = 0.014) had a higher PHQ-9 score. Compared with HCWs without loans, the PHQ-15 score of participants with loans increased by 1.10 points (95% CI: 0.13–2.06, *P* = 0.027) on average. And the PHQ-15 score of HCWs with chronic disease increased 2.62 points (95% CI: 1.38–3.86, *P* < 0.001) on average. Additionally, participants who engaged in nucleic acid collection (1.30, 95% CI: 0.26–2.33, *P* = 0.014) and who worked more than 8 h per day (0.93, 95% CI: 0.08–1.78, *P* = 0.033) had higher PHQ-15 score.

**TABLE 2:** Correlation between independent variables and scale scores estimated by generalized linear model.

Variables	GAD-7		PHQ-9		PHQ-15	
	Estimate (95% CI)	*P*-value	Estimate (95% CI)	*P*-value	Estimate (95% CI)	*P*-value
Age (per years)	0.00 (−0.04, 0.03)	0.801	0.00 (−0.04, 0.04)	0.92	−0.01 (−0.05, 0.03)	0.647
Females (vs. males)	0.38 (−0.50, 1.26)	0.394	0.06 (−0.93, 1.06)	0.899	0.82 (−0.26, 1.90)	0.138
Exercise	−0.47 (−1.27, 0.34)	0.253	−0.36 (−1.28, 0.55)	0.436	−0.83 (−1.83, 0.16)	0.100
Household income ≥10,000 per month	−0.48 (−1.20, 0.24)	0.192	−0.71 (−1.53, 0.11)	0.09	−0.65 (−1.55, 0.24)	0.153
Loans	0.16 (−0.63, 0.94)	0.693	0.57 (−0.32, 1.46)	0.207	1.10 (0.13, 2.06)	0.027
With elderly	0.92 (0.10, 1.74)	0.028	0.89 (−0.04, 1.82)	0.062	0.70 (−0.31, 1.71)	0.175
With chronic disease	1.46 (0.45, 2.46)	0.005	1.79 (0.65, 2.93)	0.002	2.62 (1.38, 3.86)	<0.001
Previous experience of fight against COVID-19	0.31 (−0.51, 1.13)	0.457	0.77 (−0.16, 1.70)	0.106	0.61 (−0.40, 1.63)	0.235
Engaged in nucleic acid collection	1.03 (0.19, 1.87)	0.016	1.20 (0.25, 2.15)	0.014	1.30 (0.26, 2.33)	0.014
Chengdong district (vs. Chengbei district)	−0.38 (−1.07, 0.31)	0.275	0.17 (−0.61, 0.96)	0.665	−0.25 (−1.11, 0.60)	0.560
Working ≥8 h per day	0.07 (−0.62, 0.76)	0.840	0.44 (−0.34, 1.23)	0.265	0.93 (0.08, 1.78)	0.033

GAD-7, Generalized Anxiety Disorder 7-Item Scale; PHQ-9, Patient Health Questionnaire-9; PHQ-15, Patient Health Questionnaire-15; CI, confidence interval; COVID-19, coronavirus disease 2019.

### Changes of anxiety, depression, and somatic symptom disorders over time and related factors

As mentioned above, of the 647 HCWs who participated in the baseline survey, 139 participants in Chengdong district completed two follow-up visits after psychological intervention. Their median age is 31 (28, 35) years old, 18.0% of them are male, and their median GAD-7 score, PHQ-9 score, and PHQ-15 score were 4 (1, 6), 4 (0, 7), and 5 (1, 9), respectively (Supplementary Table 4).

**FIGURE 2:**
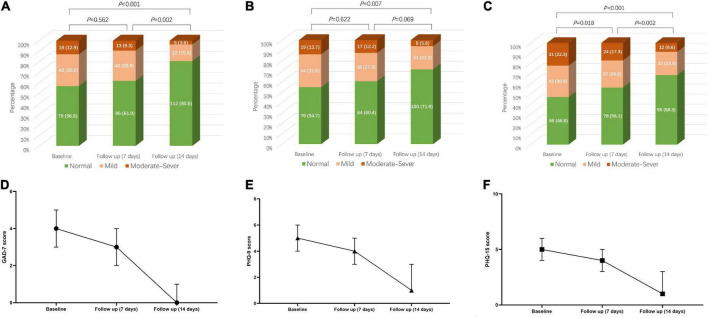
Change trend of GAD-7 score, PHQ-9 score, and PHQ-15 score over time and the change of anxiety, depression, and somatic symptom disorder severity over time based on the above scores. **(A)** Anxiety composition based on GAD-7 score at baseline, day 7 and day 14. **(B)** Depression composition based on PHQ-9 score at baseline, day 7 and day 14. **(C)** Somatic symptom disorder composition based on PHQ-9 score at baseline, day 7 and day 14. **(D)** Change trend of GAD-7 score over time. **(E)** Change trend of PHQ-9 score over time. **(F)** Change trend of PHQ-15 score over time. GAD-7, Generalized Anxiety Disorder 7-Item Scale; PHQ-9, Patient Health Questionnaire-9; PHQ-15, Patient Health Questionnaire-15.

For the HCWs who participated in the follow-up investigation, the prevalence of anxiety, depression, and somatic symptom disorders in baseline were 43.2, 45.3, and 53.2%, respectively. At the seventh day of follow-up, the prevalence of anxiety, depression, and somatic symptom disorders were 38.1, 39.6, and 43.9%, respectively, while at the 14th day of follow-up, the prevalence of anxiety, depression, and somatic symptom disorders were 19.4, 28.1, and 31.7%, respectively, which were significantly lower than the baseline (*P*_anxiety_ < 0.001, *P*_depressio*n*_ = 0.007, *P*_*rm somatic symptom disorders*_ < 0.001) (Figure 2).

Compared with the baseline, the risk of anxiety, depression, and somatic symptom disorders of HCWs decreased by 74% (OR: 0.26, 95% CI: 0.14–0.46, *P* < 0.001), 58% (OR: 0.42, 95% CI: 0.25–0.71, *P* = 0.001), and 68% (OR: 0.32, 95% CI: 0.19–0.54, *P* < 0.001), respectively at 14 days of follow-up. Physical exercise could reduce the risk of anxiety (OR: 0.34, 95% CI: 0.16–0.72, *P* = 0.005), and somatic symptom disorders (OR: 0.25, 95% CI: 0.12–0.52, *P* < 0.001) within 14 days. The risk of 14-days anxiety, depression, and somatic symptom disorders of HCWs with loans increased, and the risk of long-term anxiety and depression of healthcare workers engaging in nucleic acid increased (Table 3).

**TABLE 3:** Correlation between independent variables and anxiety, depression, and somatic disorder estimated by generalized estimation equation.

Variables	Anxiety		Depression		Somatic disorder	
	**OR (95% CI)**	***P-*value**	**OR (95% CI)**	***P*-value**	**OR (95% CI)**	***P*-value**
Time 2 vs. time 1	0.78 (0.52, 1.17)	0.226	0.76 (0.49, 1.19)	0.229	0.62 (0.41, 0.93)	0.022
Time 3 vs. time 1	0.26 (0.14, 0.46)	<0.001	0.42 (0.25, 0.71)	0.001	0.32 (0.19, 0.54)	<0.001
Age (per years)	1.04 (0.99, 1.10)	0.146	0.65 (0.35, 1.23)	0.188	1.01 (0.95, 1.06)	0.840
Females (vs. males)	0.87 (0.41, 1.86)	0.726	1.04 (0.99, 1.10)	0.097	2.20 (1.05, 4.61)	0.036
Exercise	0.34 (0.16, 0.72)	0.005	0.69 (0.36, 1.33)	0.266	0.25 (0.12, 0.52)	<0.001
Household income ≥10,000 per month	0.86 (0.49, 1.52)	0.613	0.67 (0.40, 1.13)	0.130	0.63 (0.36, 1.12)	0.116
Loans	5.87 (2.72, 12.63)	<0.001	4.63 (2.39, 8.97)	<0.001	5.50 (2.61, 11.58)	<0.001
With elderly	1.09 (0.53, 2.24)	0.813	1.06 (0.57, 1.99)	0.844	1.83 (0.98, 3.44)	0.060
With chronic disease	1.85 (0.84, 4.08)	0.124	2.07 (0.95, 4.49)	0.066	2.51 (1.01, 6.26)	0.048
Previous experience of fight against COVID-19	1.35 (0.60, 3.05)	0.468	1.07 (0.53, 2.16)	0.856	2.67 (1.24, 5.78)	0.012
Engaged in nucleic acid collection	2.89 (1.28, 6.52)	0.010	2.28 (1.03, 5.01)	0.041	1.49 (0.71, 3.14)	0.289
Working ≥8 h per day	1.15 (0.61, 2.17)	0.661	1.11 (0.62, 1.97)	0.734	1.21 (0.66, 2.20)	0.540

OR, odds ratio; CI, confidence interval; COVID-19, coronavirus disease 2019.

## Discussion

In our study, we mainly found that the prevalence of anxiety, depression, and somatic symptom disorders among HCWs fighting against COVID-19 were 46.52% (95% CI: 42.68–50.37%), 45.75% (95% CI: 41.91–49.59%), and 52.55% (95% CI: 48.70–56.40%), respectively. The results of cross-sectional study at baseline suggest that chronic diseases and engaged in nucleic acid collection were associated with increased scores of anxiety, depression, and somatic disorders. After 2 weeks of intervention, the prevalence of anxiety, depression, and somatic disorders in HCW population improved significantly. Exercise could reduce the long-term risk of anxiety, depression, and somatic symptom disorders, while loans were associated with increased risk.

Mental health disorders including mainly anxiety, depression, and somatic symptom disorders among HCWs during COVID-19 epidemics have attracted widespread attention, but the systematically and comprehensively describing the dynamic variation patterns of the psychological health evolution is not available. We performed a prospective cohort, survey-intervention study to assess the prevalence of mental health outcomes, related factors, and evaluate the changes of mental health over time and its influencing factors after psychological intervention among HCWs at high altitude during the rapid spread of the SARS-CoV-2 Omicron variant. Our findings presented a higher prevalence of anxiety, depression, and somatic symptom disorders and further indicted a direct negative correlation between mental health and regular interventions in medical health workers. Our data appear to be higher than the reported prevalence of depression (31.4%: 95% CI: 27.3–35.5%), anxiety (31.9%: 95% CI: 27.9–36.0%) among HCWs worldwide during the COVID-19 pandemic (24, 25). Our results are broadly comparable to the respective rates previously reported among HCWs during and after the MERS and SARS epidemics, in which the prevalence of depression and anxiety was high (4, 26–28). Interestingly, it should be noticed that there was an extremely high prevalence (52.55%: 95% CI: 48.70–56.40%) of somatic symptom disorders among HCWs in the online mental health assessment compared to HCWs in non-COVID times (29).

Several reasons probably account for the high prevalence of anxiety, depression, and somatic symptom disorders among HCWs. First, HCWs need to wear several layers of protective masks and clothing to meet the requirements of isolation and disinfection, which increases their work intensity and energy consumption, leading to hypoxia, and physical symptoms, such as fatigue and muscle pain. In the meantime, psychological distress may lead to somatic symptom disorders. Trauma or stressors can impair the autonomic nervous system or the stress response system (30), causing somatization or non-specific physical symptoms. Secondly, HCWs in the anti-epidemic inevitably had to contact infected persons and their secretions, resulting in high exposure risk and huge psychological pressure during the rapid spread of SARS-CoV-2 Omicron variant. Finally, the effect of oxygen deficiency in high-altitude plays an important role on mood of HCWs. High-altitude exposure with hours to a few days, triggers the onset of anxiety, depression, and somatic symptom disorders (31, 32). The decreased psychosomatic health of HCWs will also generate a negative influence on health care performance (29, 33).

The cross-sectional findings suggest that health workers with chronic diseases had higher scores of GAD-7, PHQ-9, and PHQ-15. There is a recognized association between mental and physical health. Compared with the general population, people with chronic disease have higher rates of mental health disorders, while people with mental health disorders have a greater risk of developing chronic diseases (34, 35). Of note, HCWs with loans and longer working hours were susceptible to somatic symptom disorders in our study. It has been reported that income level is related to mental health both in developed countries such as Canada and developing countries such as Turkey (36, 37). Previous data showed that working hours and shift intensity were positively correlated with mental disorders among HCWs (38, 39).

The results of generalized estimation equation based on repeated measurement data suggest that the risk of anxiety, depression, and somatic symptom disorders decreased significantly 2 weeks after psychological intervention, and physical exercise can reduce the risk of long-term anxiety and somatic disorders. The intervention in the workers with sedentary tasks confirmed the protective effect of exercise on anxiety and depression (40), while we had not found the improvement effect of exercise on depression. This may be due to our relatively short follow-up time, and the type of work of HCWs against COVID-19 was different from the above study.

The most important findings of this article confirmed that the prevalence of anxiety, depression, and somatic disorders among medical workers was significantly reduced with time, especially after 2 weeks (*P* < 0.001). Although these findings are not surprising, given that the Chengdong district is the most serious epidemic of Omicron infection, HCWs at high risk for anxiety, depression, and somatic reactive symptoms. Our data raised the question of screening for mental health disorders and risk stratification of mental health in medical staff working on frontline. Unfortunately, due to the lack of control, the change of participants’ psychological status over time in this study may be due to the change of work status or psychological intervention, which needs to be verified by subsequent studies.

Although these findings are not surprising during the epidemic of SARS-CoV-2 Omicron variant, the HCWs at high altitude had high prevalence of mental health disorders. Therefore, more attention should be paid to HCWs at high altitude, especially for those with chronic disease, loans, and those who worked with longer hours and engaged in nucleic acid collection. Although there is no sufficient evidence to suggest the role of intervention in this study, it is necessary to take appropriate measures to alleviate mental health problems for these populations.

### Limitations

Our study has several limitations. One of the major limitations of this study was the lack of a control group with HCWs who did not work on frontline against COVID-19 during the same time. Such a control group could help to validate the prevalence, risk factors, prognosis, and intervention measures of mental health disorders in HCWs on frontline. Another limitation was that, although this study yielded a large sample size, we could not decide the accurate response rate because of the nature of online survey. Additionally, selective biases may have occurred as our sample may have included more individuals who were worried about their mental health problems or those who tend to seek the required help, which may lead to overestimating the prevalence of mental disorders.

## Conclusion

At high altitude during the rapid spread of the SARS-CoV-2 Omicron variant, the prevalence of mental health disorders in HCWs is high. We also found that regular intervention measures may be protective in HCWs on frontline from mental health disorders. In the future, it may be necessary to give more support to HCWs with chronic diseases, financial burden, long working hours and relatively boring work.

## Data availability statement

The raw data supporting the conclusions of this article will be made available by the authors, without undue reservation.

## Ethics statement

The studies involving human participants were reviewed and approved by the two Institutional Review Boards (IRBs) including Qinghai Provincial People’s Hospital (Ethical number: 2022-065) and Xining Second People’s Hospital (Ethical number: 2022017). The patients/participants provided their written informed consent to participate in this study.

## Author contributions

JM and QZ had full access to all of the data in the study, took responsibility for the integrity of the data and the accuracy of the data analysis, and supervised the study. JM, XF, and CY conceptualized and designed the study. HY, KW, YX, and XZ contributed to acquisition, analysis, or interpretation of data. XF, CY, and HY drafted the manuscript. XF, CY, QZ, and JM critically revised the manuscript for important intellectual content. XF and CY contributed to the statistical analysis. All authors contributed to the article and approved the submitted version.

## Footnotes

1
https://www.r-project.org/
