# Supplementary material for: Anxiety, depression, and somatic symptom disorders in health care workers at high altitude during the rapid spread of the SARS-CoV-2 Omicron variant: A prospective cohort study
Source: Front Psychiatry. 2023 Jan 4;13:1018391. doi: 10.3389/fpsyt.2022.1018391 (PMC9846133; doi:10.3389/fpsyt.2022.1018391)
Supplement: Supplementary file 1 [file Data_Sheet_1.docx]

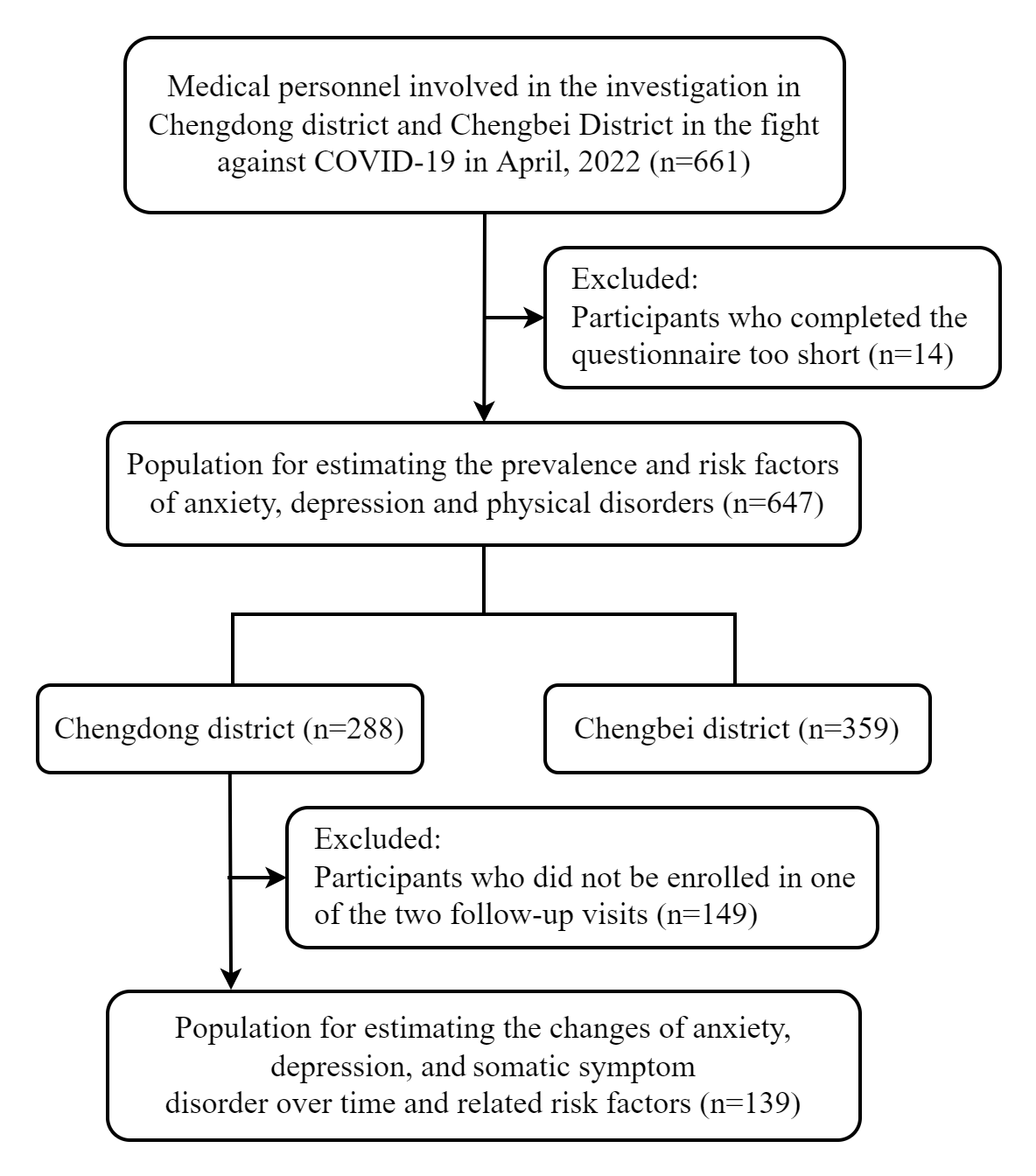


Figure S1. Flowchart of participant enrollment. Abbreviations: COVID-19, coronavirus disease 2019.

Table S1. The GAD-7scale

| Over the *last 2 weeks*, how often have you been bothered by any of the following problems? | Not at all | Several days | More than half the days | Nearly every day |
| --- | --- | --- | --- | --- |
| 1. Feeling nervous, anxious or on edge | 0 | 1 | 2 | 3 |
| 2. Not being able to stop or control worrying | 0 | 1 | 2 | 3 |
| 3. Worrying too much about different things | 0 | 1 | 2 | 3 |
| 4. Trouble relaxing | 0 | 1 | 2 | 3 |
| 5. Being so restless that it is hard to sit still | 0 | 1 | 2 | 3 |
| 6. Becoming easily annoyed or irritable | 0 | 1 | 2 | 3 |
| 7. Feeling afraid as if something awful might happen | 0 | 1 | 2 | 3 |

Table S2. The PHQ-9 scale

| Over the *last 2 weeks*, how often have you been bothered by any of the following problems? | Not at all | Several days | More than half the days | Nearly every day |
| --- | --- | --- | --- | --- |
| 1. Little interest or pleasure in doing things | 0 | 1 | 2 | 3 |
| 2. Feeling down, depressed, or hopeless | 0 | 1 | 2 | 3 |
| 3. Trouble falling or staying asleep, or sleeping too much | 0 | 1 | 2 | 3 |
| 4. Feeling tired or having little energy | 0 | 1 | 2 | 3 |
| 5. Poor appetite or overeating | 0 | 1 | 2 | 3 |
| 6. Feeling bad about yourself—or that you are a failure or have let yourself or your family down | 0 | 1 | 2 | 3 |
| 7. Trouble concentrating on things, such as reading the newspaper or watching television | 0 | 1 | 2 | 3 |
| 8. Moving or speaking so slowly that other people could have noticed? Or the opposite—being so fidgety or restless that you have been moving around a lot more than usual | 0 | 1 | 2 | 3 |
| 9. Thoughts that you would be better off dead or of hurting yourself in some way | 0 | 1 | 2 | 3 |

Table S3. The PHQ-15 scale

| During the *last 4 weeks*, how much have you been bothered by any of the following problems? | Not bothered | Bothered a little | Bothered a lot |
| --- | --- | --- | --- |
| 1. Stomach pain | 0 | 1 | 2 |
| 2. Back pain | 0 | 1 | 2 |
| 3. Pain in your arms, legs, or joints (knees, hips, etc.) | 0 | 1 | 2 |
| 4. Feeling tired or having little energy | 0 | 1 | 2 |
| 5. Trouble falling or staying asleep, or sleeping too much | 0 | 1 | 2 |
| 6. Menstrual cramps or other problems with your periods | 0 | 1 | 2 |
| 7. Pain or problems during sexual intercourse | 0 | 1 | 2 |
| 8. Headaches | 0 | 1 | 2 |
| 9. Chest pain | 0 | 1 | 2 |
| 10. Dizziness | 0 | 1 | 2 |
| 11. Fainting spells | 0 | 1 | 2 |
| 12. Feeling your heart pound or race | 0 | 1 | 2 |
| 13. Shortness of breath | 0 | 1 | 2 |
| 14. Constipation, loose bowels, or diarrhea | 0 | 1 | 2 |
| 15. Nausea, gas, or indigestion | 0 | 1 | 2 |

Table S4. Baseline characteristics of medical personnel participating in baseline survey and every follow-up in Chengdong District

| Variables | All (n=139) |
| --- | --- |
| Age (years) | 31 (28, 35) |
| Males, n (%) | 25 (18.0) |
| Married, n (%) | 99 (71.2) |
| Education, n (%) |  |
| Below bachelor degree | 12 (8.6) |
| Bachelor degree | 119 (85.6) |
| Master degree and above | 8 (5.8) |
| Nurse, n (%) | 91 (65.5) |
| Working years, n (%) |  |
| <5 years | 38 (27.3) |
| 5-9 years | 48 (34.5) |
| 10-19 years | 43 (30.9) |
| ≥20 years | 10 (7.2) |
| Title, n (%) |  |
| Primary and below | 95 (68.3) |
| Middle and above | 44 (31.4) |
| Booster injection, n (%) | 109 (78.4) |
| Household income, n (%) |  |
| <10,000 ¥ /month | 84 (60.4) |
| 10,000-19,999 ¥ /month | 48 (34.5) |
| ≥20,000 ¥ /month | 7 (5.0) |
| Loans, n (%) | 98 (70.5) |
| Live alone, n (%) | 29 (20.9) |
| With kids, n (%) | 78 (56.1) |
| With elderly, n (%) | 91 (65.5) |
| With chronic disease, n (%) | 17 (12.2) |
| Exercise, n (%) | 47 (33.8) |
| Previous experience of fight against COVID-19, n (%) | 111 (79.9) |
| Engaged in nucleic acid collection, n (%) | 107 (77.0) |
| Working hours per day, n (%) |  |
| <6h | 19 (13.7) |
| 6-7h | 69 (49.6) |
| 8-9h | 42 (30.2) |
| >9h | 9 (6.5) |
| GAD-7 score | 4 (1, 6) |
| PHQ-9 score | 4 (0, 7) |
| PHQ-15 score | 5 (1, 9) |

Data are presented as median (IQR), or n (%).Abbreviations: GAD-7, Generalized anxiety disorder 7-item Scale; PHQ-9, Patient health questionnaire-9; PHQ-15, Patient health questionnaire-15; SDS, Self-rating Depression Scale; SAS, Self-rating Anxiety Scale; COVID-19, coronavirus disease 2019; IQR, interquartile range.
